# Supplementary material for: From sequence to enzyme mechanism using multi-label machine learning
Source: BMC Bioinformatics. 2014 May 19;15:150. doi: 10.1186/1471-2105-15-150 (PMC4229970; doi:10.1186/1471-2105-15-150)
Supplement: Additional file 2 — Java code of ml2db. Additional file ml2db_code.tar.gz contains the Java source code to run the multi-label machine learning experiments and save the results to database. The code’s Javadoc is included. [file 1471-2105-15-150-S2.zip › additional file 2/ml2db/utils/doc/uk/ac/standrews/utils/main/database/DbReader.html]

DbReader


JavaScript is disabled on your browser.


- Overview
- Package
- Class
- Use
- Tree
- Deprecated
- Index
- Help

- Prev Class
- Next Class

- Frames
- No Frames

- All Classes

- Summary:
- Nested |
- Field |
- Constr |
- Method

- Detail:
- Field |
- Constr |
- Method


uk.ac.standrews.utils.main.database

## Class DbReader

- java.lang.Object
- - uk.ac.standrews.utils.main.database.DbManaged
  - - uk.ac.standrews.utils.main.database.DbReader

- Type Parameters:
  :   `K` -
  :   `V` -

  ---

    

  ```
  public class DbReader
  extends DbManaged
  ```

  Class to read from a database. For queries: if a set of queries point
  repeatedly to the same table, or you need table specific information, get a
  `TableReader` object.

  Version:
  :   20 Feb 2009

  Author:
  :   Luna De Ferrari luna.deferrari-at-ed.ac.uk

- - ### Constructor Summary

    Constructors

    Constructor and Description

    `DbReader(DbManager dbManager)`
  - ### Method Summary

    Methods

    | Modifier and Type | Method and Description |
    | --- | --- |
    | `java.sql.ResultSet` | `executeQuery(java.lang.String sql)` Executes an sql query returning a result set. |
    | `MapOfMaps` | `getColumnsValues(java.sql.ResultSet resultSet, int columnIndexOfExternalKeys, int columnIndexOfInternalKeys, int columnIndexOfValues, int expectedExternalKeysUniqueStrings, int expectedInternalKeysUniqueStrings, int expectedValuesUniqueStrings, java.lang.String defaultValue, boolean acceptEmptyStrings, java.lang.String prefix_for_column_2)` Get three columns of a `ResultSet` and store them in a [external key, internal key, value] map |
    | `java.util.AbstractCollection<java.lang.String>` | `getColumnValues(java.sql.ResultSet resultSet, int columnIndex, java.util.AbstractCollection<java.lang.String> collection, int uniqueValues)` Get the nth column of a `ResultSet` |
    | `java.sql.DatabaseMetaData` | `getDbMetaData()` |
    | `java.util.TreeSet<java.lang.String>` | `getListOfDbTables()` Gets the list of tables for a database |
    | `java.util.TreeMap<java.lang.String,java.lang.String>` | `getMap(java.lang.String sql)` Converts a query resultset into a map. |
    | `MapOfMaps` | `getMap(java.lang.String sql, int uniqueStrings, java.lang.String defaultValue, boolean acceptEmptyStrings, java.lang.String prefix_for_column_2)` Converts a query resultset into a map. |
    | `java.sql.Statement` | `getStatement()` |
    | `java.util.AbstractCollection<java.lang.String>` | `queryResultsColumnToCollection(java.lang.String sql, int columnIndex, java.util.AbstractCollection<java.lang.String> collection, int uniqueValues)` From two resultset columns to a key-value map |
    | `java.util.TreeSet<java.lang.String>` | `queryResultsColumnToSet(java.lang.String sql, int columnIndex, int uniqueValues)` Reduces a query results column to a set (beware: it eliminates duplicates!) |
    | `java.util.Vector<java.lang.String>` | `queryResultsColumnToVector(java.lang.String sql, int columnIndex, int uniqueValues)` |
    | `boolean` | `tableExistsInDb(java.lang.String tableName)` Checks if a table exists in the database |

    - ### Methods inherited from class uk.ac.standrews.utils.main.database.DbManaged

      `getDbConnection, getDbManager, getDbReader`
    - ### Methods inherited from class java.lang.Object

      `equals, getClass, hashCode, notify, notifyAll, toString, wait, wait, wait`

- - ### Constructor Detail


    - #### DbReader

      ```
      public DbReader(DbManager dbManager)
      ```
  - ### Method Detail


    - #### executeQuery

      ```
      public java.sql.ResultSet executeQuery(java.lang.String sql)
      ```

      Executes an sql query returning a result set.

      Parameters:
      :   `sql` - an SQL query

      Returns:
      :   a `ResultSet` with the query results

      Throws:
      :   `java.sql.SQLException`


    - #### getColumnsValues

      ```
      public MapOfMaps getColumnsValues(java.sql.ResultSet resultSet,
                               int columnIndexOfExternalKeys,
                               int columnIndexOfInternalKeys,
                               int columnIndexOfValues,
                               int expectedExternalKeysUniqueStrings,
                               int expectedInternalKeysUniqueStrings,
                               int expectedValuesUniqueStrings,
                               java.lang.String defaultValue,
                               boolean acceptEmptyStrings,
                               java.lang.String prefix_for_column_2)
      ```

      Get three columns of a `ResultSet` and store them in a [external
      key, internal key, value] map

      Parameters:
      :   `resultSet` -
      :   `columnIndexOfExternalKeys` - column where external keys are (columns index starting at 0)
      :   `columnIndexOfInternalKeys` - column where internal keys are (columns index starting at 0)
      :   `columnIndexOfValues` - column where the value are (columns index starting at 0)
      :   `map` - the map to put the [external key, internal key, value]
      :   `expectedExternalKeysUniqueStrings` - the expected number of \*unique\* string values among the
          external keys \* @param expectedInternalKeysUniqueStrings the
          expected number of \*unique\* string values among the internal
          keys
      :   `defaultValue` - the default value for column 3 if the result set only has 2
          columns

      Returns:
      :   a map containing the result set data as keys and values of a map


    - #### getColumnValues

      ```
      public java.util.AbstractCollection<java.lang.String> getColumnValues(java.sql.ResultSet resultSet,
                                                                   int columnIndex,
                                                                   java.util.AbstractCollection<java.lang.String> collection,
                                                                   int uniqueValues)
      ```

      Get the nth column of a `ResultSet`

      Parameters:
      :   `resultSet` -
      :   `columnIndex` - the column number (starting at 0)
      :   `uniqueValues` - the number of unique values likely to exist in the data (to
          create a string pool)

      Returns:
      :   the column values


    - #### getDbMetaData

      ```
      public java.sql.DatabaseMetaData getDbMetaData()
      ```


    - #### getListOfDbTables

      ```
      public java.util.TreeSet<java.lang.String> getListOfDbTables()
      ```

      Gets the list of tables for a database

      Returns:
      :   a list of table names


    - #### getMap

      ```
      public java.util.TreeMap<java.lang.String,java.lang.String> getMap(java.lang.String sql)
      ```

      Converts a query resultset into a map. Beware: Null values are discarded
      both as keys and as values.

      Parameters:
      :   `sql` - the sql query (the first column should be the key and the
          second column the value)

      Returns:
      :   the map with the resultset results


    - #### getMap

      ```
      public MapOfMaps getMap(java.lang.String sql,
                     int uniqueStrings,
                     java.lang.String defaultValue,
                     boolean acceptEmptyStrings,
                     java.lang.String prefix_for_column_2)
      ```

      Converts a query resultset into a map. Beware: Null values are discarded
      both as keys and as values.

      Parameters:
      :   `sql` - the sql query
      :   `columnIndexOfKeys` - the index for the results column which contains the keys (eg.
          0)
      :   `columnIndexOfValues` - the index for the results column which contains the values
          (eg. 1)
      :   `defaultForValues` - if the query only returns two columns (external and internal
          keys), this default value is used to fill all tuples [external
          key, internal key, value]

      Returns:
      :   return a map of maps (to each key corresponds an internal map of
          keys and values)


    - #### getStatement

      ```
      public java.sql.Statement getStatement()
      ```


    - #### queryResultsColumnToCollection

      ```
      public java.util.AbstractCollection<java.lang.String> queryResultsColumnToCollection(java.lang.String sql,
                                                                                  int columnIndex,
                                                                                  java.util.AbstractCollection<java.lang.String> collection,
                                                                                  int uniqueValues)
      ```

      From two resultset columns to a key-value map

      Parameters:
      :   `sql` -
      :   `columnIndexOfKeys` -
      :   `columnIndexOfValues` -
      :   `map` -
      :   `expectedNumberOfKeysUniqueStrings` -
      :   `expectedNumberOfValuesUniqueStrings` -

      Returns:
      :   a map with two of the resultset columns as keys and values
          respectively
          public OneToManyMap queryResultsColumnsToMap(String sql,
          int columnIndexOfKeys, int columnIndexOfValues, int
          expectedNumberOfKeysUniqueStrings, int
          expectedNumberOfValuesUniqueStrings) { if (m\_debug) {
          LogUtils.log("Query started: " + sql); } ResultSet rs =
          this.executeQuery(sql); if (m\_debug) {
          LogUtils.log("Query ended: " + sql); } if (rs != null) {
          OneToManyMap map = new OneToManyMap(); return
          this.get2ColumnsValues(rs, columnIndexOfKeys,
          columnIndexOfValues, map, expectedNumberOfKeysUniqueStrings,
          expectedNumberOfValuesUniqueStrings); } else {
          LogUtils.error(this, "Result set is null."); return null; }
          }


    - #### queryResultsColumnToSet

      ```
      public java.util.TreeSet<java.lang.String> queryResultsColumnToSet(java.lang.String sql,
                                                                int columnIndex,
                                                                int uniqueValues)
      ```

      Reduces a query results column to a set (beware: it eliminates
      duplicates!)

      Parameters:
      :   `sql` - a query to extract the columns from database
      :   `columnIndex` - the index of the column of interest

      Returns:
      :   the set of column results


    - #### queryResultsColumnToVector

      ```
      public java.util.Vector<java.lang.String> queryResultsColumnToVector(java.lang.String sql,
                                                                  int columnIndex,
                                                                  int uniqueValues)
      ```


    - #### tableExistsInDb

      ```
      public boolean tableExistsInDb(java.lang.String tableName)
      ```

      Checks if a table exists in the database

      Parameters:
      :   `tableName` -

      Returns:
      :   true if a table with the given name exists in the database


- Overview
- Package
- Class
- Use
- Tree
- Deprecated
- Index
- Help

- Prev Class
- Next Class

- Frames
- No Frames

- All Classes

- Summary:
- Nested |
- Field |
- Constr |
- Method

- Detail:
- Field |
- Constr |
- Method
